# Drinking to Cope is Uniquely Associated with Less Specific and Bleaker Future Goal Generation in Young Hazardous Drinkers

**DOI:** 10.1007/s10862-023-10032-0

**Published:** 2023-02-27

**Authors:** Ruichong Shuai, Bella Magner-Parsons, Lee Hogarth

**Affiliations:** School of Psychology, University of Exeter, Washington Singer Building, Perry Road, EX4 4QG Exeter, UK

**Keywords:** Goal specificity, Drinking to cope, Mental health problems, Substance use problems

## Abstract

Groups with mental health and/or substance use problems generate less detailed descriptions of their future goals. As substance use to cope with negative affect is common to both groups, this characteristic might be uniquely associated with less specific goal descriptions. To test this prediction, 229 past year hazardous drinking undergraduates aged 18–25 years wrote about three positive future life goals in an open-ended survey, before reporting their internalizing (anxiety and depression) symptoms, alcohol dependence severity and motivations for drinking: coping, conformity, enhancement and social. Future goal descriptions were experimenter-rated for detail specificity, and participant-self-rated for positivity, vividness, achievability, and importance. Effort in goal writing was indexed by time spent writing and total word count. Multiple regression analyses revealed that drinking to cope was uniquely associated with the production of less detailed goals, and lower self-rated positivity and vividness of goals (achievability and importance were also marginally lower), over and above internalizing symptoms, alcohol dependence severity, drinking for conformity, enhancement and social motives, age, and gender. However, drinking to cope was not uniquely associated with reduced effort in writing goals: time spent and word count. In sum, drinking to cope with negative affect is a unique characteristic predicting the generation of less detailed and bleaker (less positive and vivid) future goals, and this is not due to lower effort in reporting. Future goal generation may play a role in the aetiology of comorbidity of mental health and substance use problems, and therapeutic targeting of goal generation might benefit both conditions.

**Supplementary Information:**

The online version contains supplementary material available at 10.1007/s10862-023-10032-0.

## Introduction

The representation of personal goals plays a fundamental role in motivating human behaviour (Lunenburg, 2011) and differences in this may play a role in various psychological conditions (Hallford et al., 2018; Moustafa et al., 2019). Individuals with depression, for instance, provide less specific descriptions of their future goals when asked (Gamble et al., 2019). For instance, Dickson and Moberly (2013) asked 21 participants with current major depression and 24 healthy controls to write down as many specific and discrete future goals that they could think of in 90 s. Goal specificity was coded by two blinded raters who coded each goal as specific if it contained a particular target feature, objective or reference to time, place, or person (e.g., ‘to finish completing the personal development review forms this evening’), or coded each goal as general if it referred only to a global aspiration (e.g., ‘to be happy’). The depressed group reported a smaller number of specific goals than the healthy controls, suggesting this characteristic may play a role in the aetiology of depression and may be a useful target for intervention.

Similarly to individuals with depression, individuals with substance use problems also report less detailed future events and goals, suggesting this characteristic may be transdiagnostic. For instance, Mercuri et al. (2015) conducted semi-structured autobiographical interviews with chronic opiate users where they described a previously experienced event (control) or a novel future event they were likely to experience. Three blinded raters quantified the interview transcripts by counting the number of episodic versus non-episodic details (such as repetitions, semantic, and tangential information) for both the past and future events. Compared to healthy controls, the opiate users reported less specific future events but comparable specificity of past events. Similarly, El Haj et al. (2019) interviewed individuals with alcohol use disorder who described one past and one future event and counted the number of details using a similar coding scheme to Dickson and Moberly (2013). Compared to healthy controls, individuals with alcohol use disorder showed reduced specificity for both the past and future events, suggesting a more general reduction in specificity of episodic thinking. Another three studies have shown that individuals with alcohol use disorder generated less detailed descriptions of future events compared to healthy controls (Mercuri et al., 2016; Nandrino & El Haj, 2019; Noël et al., 2022), although one study has reported a null difference (Moustafa et al., 2018). Finally, Mercuri et al. (2018) observed reduced specificity of future event descriptions in regular but not recreational cannabis users compared to controls, suggesting that this characteristic may increase with dependence severity.

Given that mental health and substance use problems are comorbid (Foulds et al., 2015; Najt et al., 2011), it is unclear which condition is uniquely associated with reduced specificity of future goal/event descriptions. To elaborate, in the two studies by Mercuri et al. (2015, 2016) noted above, the opiate/control group comparison was confounded by higher anxiety and depression symptoms in the opiate group, so either substance use or mental health status could be linked to reduced specificity of future event descriptions. Moreover, in the study by El Haj et al. (2019), depression severity within the alcohol dependent sample was correlated with less specific future event descriptions, raising uncertainty about which symptom type was uniquely linked to future event specificity. To resolve this confounding issue, studies are needed to isolate the unique associations that mental health and substance use severity have with reduced specificity of future goal/event descriptions. In addition to testing these unique associations, the current study also tested the novel possibility that self-reported substance use to cope with negative affect may be uniquely linked to reduced specificity of personal goals. The basis for this claim is that self-reported use of substances to cope with negative affect is common to individuals with mental health and substance use problems (Anker et al., In press; Menary et al., 2011; Mohr et al., 2018; Young-Wolff et al., 2009), which may explain why both groups show reduced specificity of future goal/event descriptions.

The current study tested whether reduced specificity of future goal descriptions is uniquely associated with internalizing symptoms (anxiety/depression), alcohol dependence severity or coping motives in the past year hazardous drinking undergraduates. In an online survey, participants wrote about three positive future goals in open ended text boxes with no time limit, and self-rated each goal for positivity, vividness, achievability and importance. The specificity of each goal was coded by two blinded raters using a modified coding scheme based on previous studies (Dickson & MacLeod, 2004; Dickson & Moberly, 2013). The duration of time spent writing the goals and the total word count quantified effort in the task (making seven indices of goal generation in total). Then, participants completed questionnaires measuring internalizing symptoms, alcohol use disorder severity, drinking motives (coping, conformity, enhancement and social ), age and gender. Correlation and multiple regression analyses tested the bivariate and unique associations that these questionnaire indices had with each of the seven indices of goal generation. These exploratory analyses attempted to address two research questions which have not been explored before in the literature: the foremost question was which questionnaire characteristic would show the strongest unique association with reduced specificity of the goal description, addressing the confounding problem revealed by past literature. Secondary questions were whether questionnaire characteristics would be uniquely associated with self-reported positivity, vividness, achievability and importance of goals, and/or with reduced effort expended in the goal writing task.

## Methods

### Participants

Participants were recruited from the Psychology research pool at Exeter and the Facebook page “Overheard at Exeter”. A total of 426 participants completed the set of measures, from which 229 were selected on the basis of being aged 18–25 who reported past year hazardous drinking. Hazardous drinking was defined by total score of ≥ 3 on the Alcohol Use Disorders Identification Test, as the minimal criterion validated in international samples (Nadkarni et al., 2019). Restriction of the analytical sample ensured that the theoretical model derived could be applied to young adults who are at risk of future alcohol problems. The analytical sample had a mean age of 19.83 (SD = 1.61) and compromised 84% females. Participants provided informed consent, were debriefed and reimbursed with course credits or a £3 Amazon voucher depending on their wishes. The study was approved by the School of Psychology Research Ethics Committee.

### Questionnaires

On Qualtrics online survey platform, participants reported their age and gender after reading the information sheet and completing the consent form. Participants then completed a goal writing task adapted from a previous study (O’Neill et al., 2016). The following text was presented to participants for instruction on describing future goals: ‘Please describe 3 positive goals that you want to achieve in about 3 weeks. For example, goals that are related to hobbies, volunteering, acquiring new skills or exercise (the goals should NOT relate to food or alcohol etc.). Please start with the goal you feel is most important to you’. Instruction on non-food or-alcohol related goals was to ensure that participants would not associate positive future goal generation with reinforcement or craving related to food/substances (i.e., to encourage abstinence goals). Next, participants were presented with a blank box to type in their first goal with instruction as ‘Please describe your first goal, being as detailed as possible, and imagining how you will feel having achieved it in the space below’. Participants were then asked to rate their first goal for positivity, vividness, achievability, and importance on a 9-point scale, ranging from 1 (not at all) to 9 (very). The same procedure was followed for the second and third goals. The duration spent generating each goal and the word count for each goal were recorded and averaged across three goals for analysis.

The **Patient Health Questionnaire Depression Scale** (PHQ-8, Kroenke et al., 2009) and the **Generalized Anxiety Disorder Questionnaire** (GAD-7, Löwe et al., 2008) were used to measure internalizing symptoms. The PHQ-8 contains eight items (e.g., ‘little interest or pleasures in doing things’) and the GAD-7 contains 7 items (e.g., ‘feeling nervous, anxious or on edge’), which participants endorsed on a scale from 0 ‘*Not at all*’ to 3 ‘*Nearly every day*’. The two scale mean scores were strongly correlated (*r* = .80, *p* < .001), so they were averaged to create a single score for internalizing symptoms. A score of 10 marks the boundary between mild and moderate symptom severity.

The **Alcohol Use Disorder Identification Test** (AUDIT) containing 10 items was used to assess alcohol consumption (e.g. ‘How often do you have a drink containing alcohol’) and alcohol problems (e.g., ‘How often during the last year have you found that you were not able to stop drinking once you had started’) over the past 12 months (validated by Babor et al., 2001). The total score can range from 0 to 40, which was used in the final analysis to indicate alcohol dependence severity (hereafter ‘alcohol dependence’).

The **Drinking Motives Questionnaire Revised** (DMQR validated by Grant et al., 2007) contains 28 items describing reasons which might motivate participants to drink, which they endorse on a scale ranging from 0 ‘never’ to 10 ‘always’. From these, five subscales were calculated assessing drinking to cope with anxiety (e.g. ‘to relax’), and to cope with depression (e.g. ‘to numb my pain’), drinking for pleasure enhancement (e.g. ‘to get a high’), for conformity (e.g. ‘to be liked’), and to be social (e.g. ‘as a way to celebrate’). The coping with anxiety/depression subscales were averaged to create a single ‘drinking to cope’ score because they were highly correlated (*r* = .75, *p* < .001).

### Analytical Plan

A coding scheme modified from previous studies was used to categorize goal specificity (Dickson & MacLeod, 2004; Dickson & Moberly, 2013). Each goal was coded as specific (scored as 2) if it described a future aspiration with a particular target feature plus a reference to time, place, or people (e.g., ‘to get a job after my degree’), or coded as moderate (scored as 1) if it included a specific target feature but no reference to time, place or people (e.g., ‘to get above 60% in Cognition and Development module’), or coded as general (scored as 0) if it referred to a global or abstract aspiration rather than a specific target feature (e.g., ‘to read more’). The full coding scheme can be found in supplemental materials. Two independent raters were blinded to other outcome measures (e.g., drinking motives). Differences on rating were firstly discussed between two raters for an agreement, however, if no agreement was reached, the primary rater (i.e., the main experimenter of the study) would make a decision on the final rating. The inter-rater reliability between two independent raters for goal specificity across three goals was calculated on a representative subsample size of 24.5% of the sample and yielded a Krippendorff’s alpha > 0.94 indicating good consistency of coding (Lombard et al., 2002). The experimenter-rated specificity codes were summed across all three goals, so could range from 3 to 9 with higher scores reflecting greater goal specificity.

IBM SPSS Statistics version 28 was used for data analysis. Univariate outliers (> 1.5 times the interquartile range) were winsorized to match the nearest non-outlying score to ensure that correlation and regression analyses were not unduly influenced by outliers. Assumptions for multiple regression models were checked and were met with respect to no multicollinearity (indicated by VIF scores < 10), independence of residuals (indicated by Durbin-Watson statistic values around 2), and no influential cases biasing the models (indicated by Cook’s distance < 1). Assumption of homoscedasticity was tested by Spearman’s correlations between standardized predicted values and standardized absolute residuals and was met by most regression models (indicated by non-significant Spearman’s correlations), except for mean time spent writing each goal. Finally, total word count and mean time spent writing each goal violated the assumption of normal distribution of residuals. These violations may increase the possibility of false positive for multiple regression models with word count and writing time as the outcomes.

A Pearson bivariate correlation matrix tested the unadjusted relationships between all the variables. Then a separate multiple regression model was run with each index of goal generation as the outcome: experimenter-rated goal specificity, participant-rated goal positivity, vividness, achievability, and importance, mean time spent writing each goal and total word count. Each regression model contained all of the predictor variables: internalizing symptoms, alcohol dependence, coping motives, conformity motives, enhancement motives, social motives, age and gender.

## Results

### Participants

Descriptive data (mean and standard deviation) were reported in Table 1. On average, the sample fell below the cut-point for mild to moderate internalizing symptom severity (i.e., ≤ 10 in the PHQ and GAD), but fell above the cut-point for hazardous drinking (i.e., ≥ 3 in the AUDIT, validated by Nadkarni et al., 2019). Participants scored higher on drinking for enhancement and socialising, compared to drinking to cope and for conformity. Regarding experimenter-coded specificity, there was a roughly even split between specific goals and non-specific (general and moderate) goals. Participants reported a similar level of positivity, vividness, achievability and importance of goals.

### Bivariate Correlation Coefficients

Table 1 showed bivariate unadjusted correlations between variables. Coping motives were significantly associated with reduced specificity, positivity and achievability of goals, and lower total word count. By contrast, internalizing symptoms were only associated with reduced goal achievability. Alcohol dependence was not associated with any index of goal generation. The other drinking motives showed some significant correlations with indices of goal generation, but most were non-significant in the multiple regression analyses as reported below. Finally, self-rated evaluations of goals were all inter-correlated, but not with experimenter-coded specificity, writing time or word count, which were themselves inter-correlated. Age did not correlate with indices of goal generation, and gender only correlated with writing time with females writing for a shorter length of time. After applying Bonferroni Correction for multiple testing (*p*-value was adjusted to 0.05/15 = 0.0033), only the correlation between coping motives and reduced specificity (*p* < .001) and the correlation between gender and writing time (*p* = .001) were significant. However, unique associations between variables were tested in multiple regression models reported below, where multiple variables were controlled simultaneously to test partial regression coefficients with less risk of Type I Error resulted from multiple comparisons (Menon, 2019).

**Table 1:** Bivariate Pearson correlations between EFT indices, internalizing problems, alcohol dependence and drinking motives.

Measures	Internalizing problems	Alcohol dependence	Coping motives	Conformity motives	Enhancement motives	Social motives	Age	Gender	Experimenter-coded goal specificity	Self-rated positivity	Self-rated vividness	Self-rated achievability	Self-rated importance	Mean time spent writing each goal	Total word count	Mean (SD, range)
Internalizing symptoms	(0.94)															8.40 (5.46, 0-22.5)
Alcohol dependence	0.18**	(0.64)														10.07 (4.52, 4–22)
Coping motives	0.43***	0.37***	(0.95)													3.64 (2.34, 0-9.89)
Conformity motives	0.20**	0.24***	0.44***	(0.88)												2.21 (2.19, 0-8.4)
Enhancement motives	0.14*	0.37***	0.54***	0.19**	(0.85)											6.05 (2.16, 0-9.8)
Social motives	0.18**	0.33***	0.41***	0.30***	0.62***	(0.82)										7.28 (1.70, 3–10)
Age	-0.04	-0.17*	-0.07	-0.07	-0.07	-0.09	-									19.83 (1.61, 18–25)
Gender	0.18**	-0.01	0.07	0.02	0.04	0.07	-0.19**	-								M/F (35/190)
Experimenter-coded goal specificity	-0.09	-0.02	-0.23***	-0.09	-0.16*	-0.07	-0.06	-0.08	-							General (13.83%), Moderate (33.04%), Specific (52.84%)
Self-rated positivity	-0.03	-0.01	-0.15*	-0.18**	0.08	0.01	0.02	0.10	-0.01	(0.66)						7.79 (0.92, 5–9)
Self-rated vividness	0.02	0.01	-0.12	-0.08	-0.05	-0.08	0.06	0.06	0.002	0.50***	(0.59)					6.96 (1.03, 4.4-9)
Self-rated achievability	-0.15*	0.01	-0.16*	-0.09	-0.02	-0.08	0.10	0.08	0.12	0.34***	0.47***	(0.55)				7.23 (0.98, 5–9)
Self-rated importance	0.06	0.04	-0.10	-0.07	-0.06	-0.04	0.10	0.12	0.02	0.34***	0.26***	0.17*	(0.25)			7.13 (1.04. 5–9)
Mean time spent writing each goal	-0.09	-0.09	-0.08	-0.14*	-0.04	-0.10	0.12	-0.22**	0.27***	-0.09	-0.05	-0.03	0.04	-		1.66 (1.06, 0.14-4)
Total word count	-0.05	-0.07	-0.14*	-0.15*	-0.12	-0.09	0.04	0.06	0.44***	-0.03	0.02	0.10	0.05	0.63***	-	115.24 (71.47, 10–298)

_*Note*. Gender ratio was reported as two categories for gender, due to no insufficient data in the categories of “Other” and “Prefer not to say” for meaningful statistics. Cronbach’s alpha reliability statistics for measures were reported diagonally in the table. Significant correlations before Bonferroni correction are emboldened, *=*p*<.05, **=*p*<.01, ***=*p*<.001._

**Fig 1.**
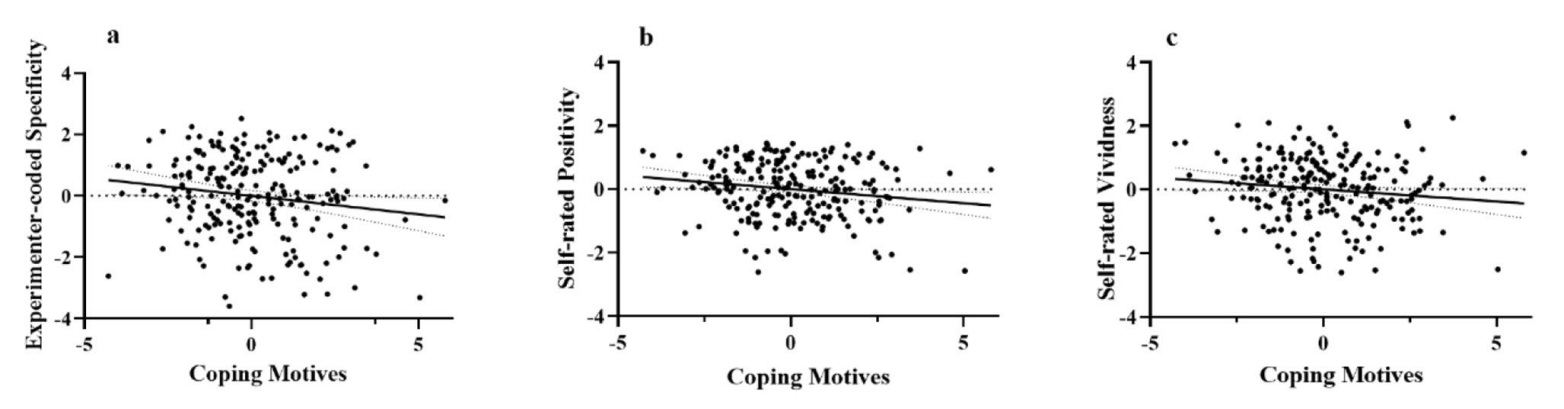
a, b and c show that coping motives were uniquely correlated with reduced experimenter-coded goal specificity, self-rated positivity and vividness, respectively.

### Multiple Regression Models

Multiple regression analyses (see Tables 2, 3, 4, 5, 6, 7 and 8) indicated that coping motives were uniquely and significantly associated with reduced experimenter-rated goal specificity (see Fig. 1a), over internalizing symptoms, alcohol dependence severity, other drinking motives, age and gender. In addition, coping motives were uniquely significantly associated with reduced self-rated positivity and vividness of goals (see Fig. 1b and 1c), and marginally with reduced achievability and importance of goals. Enhancement motives only showed a unique association with greater goal positivity (in the opposite direction to coping motives). Gender showed unique associations with indices of goal generation, such that females rated their goals as more achievable and important but spent less time writing their goals. No unique associations were found with internalizing symptoms, alcohol dependence severity, other drinking motives and age in relation to any of the indices of goal generation.

**Table 2:** Multiple regression model for predicting experimenter-coded goal specificity

Model statistics: *R*^*2*^ = 8.0%, adjusted *R*^*2*^ = 4.6%, *F* = 2.34, *p* = .020						
Variables	Unstandardized β	Standardized β	t	*p*	Partial r	Partial R^2^ (%)
Internalizing symptoms	0.002	0.01	0.13	0.899	0.01	0.008
Alcohol dependence	0.02	0.07	0.91	0.367	0.06	0.37
**Coping motives**	**-0.13**	**-0.21**	**-2.32**	**0.021**	**-0.16**	**2.43**
Conformity motives	-0.02	-0.03	-0.37	0.712	-0.03	0.06
Enhancement motives	-0.08	-0.13	-1.42	0.158	-0.10	0.92
Social motives	0.05	0.06	0.74	0.458	0.05	0.26
Age	-0.06	-0.07	-1.06	0.289	-0.07	0.52
Gender	-0.29	-0.08	-1.11	0.268	-0.08	0.56

_*Note*. Significant predictors are emboldened. Partial r = correlation coefficient when controlled for other variables in the model. Partial R_^2^_= the partial variance (%) explained by the predictors when controlling for all other variables in the model._

**Table 3:** Multiple regression model for predicting self-rated positivity

Model statistics: *R*^*2*^ = 9.1%, adjusted *R*^*2*^ = 5.7%, *F* = 2.70, *p* = .008						
Variables	Unstandardized β	Standardized β	t	*p*	Partial r	Partial R^2^ (%)
Internalizing symptoms	0.01	0.05	0.70	0.488	0.05	0.22
Alcohol dependence	0.01	0.03	0.43	0.665	0.03	0.08
**Coping motives**	**-0.10**	**-0.25**	**-2.77**	**0.006**	**-0.19**	**3.42**
Conformity motives	-0.05	-0.13	-1.77	0.079	-0.12	1.42
**Enhancement motives**	**0.10**	**0.23**	**2.51**	**0.013**	**0.17**	**2.86**
Social motives	-0.002	-0.004	-0.05	0.964	-0.003	0.001
Age	0.01	0.01	0.21	0.833	0.01	0.02
Gender	0.26	0.10	1.53	0.127	0.10	1.08

_*Note*. Significant predictors are emboldened. Partial r = correlation coefficient when controlled for other variables in the model. Partial R_^2^_= the partial variance (%) explained by the predictors when controlling for all other variables in the model._

**Table 4:** Multiple regression model for predicting self-rated vividness

Model statistics: *R*^*2*^ = 4.8%, adjusted *R*^*2*^ = 1.3%, *F* = 1.37, *p* = .213						
Variables	Unstandardized β	Standardized β	t	*p*	Partial r	Partial R^2^ (%)
Internalizing symptoms	0.01	0.07	0.98	0.328	0.07	0.45
Alcohol dependence	0.02	0.10	1.33	0.184	0.09	0.81
**Coping motives**	**-0.09**	**-0.21**	**-2.23**	**0.027**	**-0.15**	**2.25**
Conformity motives	-0.01	-0.02	-0.22	0.825	-0.02	0.02
Enhancement motives	0.04	0.08	0.79	0.428	0.05	0.29
Social motives	-0.05	-0.09	-0.97	0.334	-0.07	0.44
Age	0.06	0.09	1.25	0.212	0.09	0.72
Gender	0.24	0.09	1.25	0.212	0.09	0.72

_*Note*. Significant predictors are emboldened. Partial r = correlation coefficient when controlled for other variables in the model. Partial R_^2^_= the partial variance (%) explained by the predictors when controlling for all other variables in the model._

**Table 5:** Multiple regression model for predicting self-rated achievability

Model statistics: *R*^*2*^ = 7.0%, adjusted *R*^*2*^ = 3.6%, *F* = 2.03, *p* = .044						
Variables	Unstandardized β	Standardized β	t	*p*	Partial r	Partial R^2^ (%)
Internalizing symptoms	-0.02	-0.11	-1.47	0.144	-0.10	0.98
Alcohol dependence	0.02	0.10	1.39	0.167	0.09	0.88
Coping motives	-0.07	-0.16	-1.76	0.079	-0.12	1.42
Conformity motives	-0.004	-0.01	-0.11	0.914	-0.01	0.005
Enhancement motives	0.05	0.11	1.18	0.238	0.08	0.64
Social motives	-0.05	-0.09	-1.01	0.313	-0.07	0.48
Age	0.08	0.13	1.86	0.065	0.13	1.56
**Gender**	**0.37**	**0.14**	**1.98**	**0.049**	**0.13**	**1.77**

_*Note*. Significant predictors are emboldened. Partial r = correlation coefficient when controlled for other variables in the model. Partial R_^2^_= the partial variance (%) explained by the predictors when controlling for all other variables in the model_

**Table 6:** Multiple regression model for predicting self-rated importance

Model statistics: *R*^*2*^ = 5.9%, adjusted *R*^*2*^ = 2.4%, *F* = 1.69, *p* = .102						
Variables	Unstandardized β	Standardized β	t	*p*	Partial r	Partial R^2^ (%)
Internalizing symptoms	0.02	0.09	1.19	0.235	0.08	0.66
Alcohol dependence	0.03	0.13	1.70	0.092	0.12	1.32
Coping motives	-0.07	-0.17	-1.79	0.075	-0.12	1.46
Conformity motives	-0.02	-0.03	-0.43	0.667	-0.03	0.08
Enhancement motives	0.001	0.002	0.02	0.987	0.001	0.001
Social motives	-0.01	-0.02	-0.17	0.862	-0.01	0.01
Age	0.08	0.13	1.86	0.065	0.13	1.56
**Gender**	**0.41**	**0.14**	**2.10**	**0.037**	**0.14**	**1.99**

_*Note*. Significant predictors are emboldened. Partial r = correlation coefficient when controlled for other variables in the model. Partial R_^2^_= the partial variance (%) explained by the predictors when controlling for all other variables in the model._

**Table 7:** Multiple regression model for predicting goal duration

Model statistics: *R*^*2*^ = 8.1%, adjusted *R*^*2*^ = 4.7%, *F* = 2.38, *p* = .018						
Variables	Unstandardized β	Standardized β	t	*p*	Partial r	Partial R^2^ (%)
Internalizing symptoms	-0.004	-0.02	-0.24	0.807	-0.02	0.03
Alcohol dependence	-0.01	-0.05	-0.73	0.469	-0.05	0.24
Coping motives	0.003	0.01	0.08	0.934	0.01	0.004
Conformity motives	-0.06	-0.12	-1.63	0.106	-0.11	1.21
Enhancement motives	0.02	0.05	0.51	0.614	0.03	0.12
Social motives	-0.04	-0.07	-0.82	0.416	-0.06	0.30
Age	0.05	0.07	1.04	0.299	0.07	0.50
**Gender**	**-0.57**	**-0.20**	**-2.88**	**0.004**	**-0.19**	**3.69**

_*Note*. Significant predictors are emboldened. Partial r = correlation coefficient when controlled for other variables in the model. Partial R_^2^_= the partial variance (%) explained by the predictors when controlling for all other variables in the model._

**Table 8:** Multiple regression model for predicting total word count

Model statistics: *R*^*2*^ = 4.4%, adjusted *R*^*2*^ = 0.8%, *F* = 1.23, *p* = .282						
Variables	Unstandardized β	Standardized β	t	*p*	Partial r	Partial R^2^ (%)
Internalizing symptoms	-0.01	0	-0.01	0.996	0	0
Alcohol dependence	0.14	0.01	0.12	0.907	0.01	0.006
Coping motives	-2.20	-0.07	-0.76	0.446	-0.05	0.27
Conformity motives	-3.52	-0.11	-1.42	0.157	-0.10	0.92
Enhancement motives	-2.59	-0.08	-0.82	0.412	-0.06	0.31
Social motives	0.13	0.003	0.03	0.973	0.002	0.0004
Age	2.13	0.05	0.69	0.490	0.05	0.22
Gender	15.45	0.08	1.13	0.259	0.08	0.59

_*Note.* Significant predictors are emboldened. Partial r = correlation coefficient when controlled for other variables in the model. Partial R_^2^_= the partial variance (%) explained by the predictors when controlling for all other variables in the model._

## Discussion

To the authors’ knowledge, this is the first study to investigate whether descriptions of personal future goals would uniquely differ as a function of internalizing symptoms, alcohol dependence severity, and drinking to cope with negative affect. The findings confirmed the novel proposal that drinking to cope could be the strongest unique predictor of reduced experimenter-rated goal specificity. In addition, drinking to cope uniquely and significantly predicted reduced self-rated goal positivity and vividness, and marginally predicted reduced self-rated goal achievability and importance. These findings suggest that drinking to cope is uniquely associated with less specific and bleaker future goals, above internalizing and alcohol dependence symptoms. Importantly, drinking to cope was not uniquely associated with reduced effort in the writing task indexed by writing time or word count, suggesting that low effort cannot readily explain the associations. By contrast, internalizing symptoms or alcohol dependence severity showed no unique associations with goal generation (contradicting published literature – see below). Unexpectedly, drinking for enhancement was uniquely associated with more positive self-rating of goals, in the opposite direction to drinking to cope. Finally, compared to males, females were more likely to find their goals more achievable and more important and spent less time writing their goals.

The studies reviewed in the introduction consistently demonstrated that descriptions of future goals or events were less detailed in groups with depression and/or substance use disorders compared to healthy controls (Dickson & Moberly, 2013; Mercuri et al., 2015, 2018). By contrast, the current study did not find any significant associations between goal specificity and internalizing symptoms or alcohol dependence severity. There was only a weak bivariate association between self-rated achievability of future goals and internalizing symptoms, but this was not significant in multiple regression and so the association was not unique. This discrepancy with past studies is presumably due to them having recruited clinical samples that met diagnostic criteria for the disorder under investigation, creating a starker contrast in severity between the experimental and control groups than was provided by natural variation in our opportunistically recruited sub-clinical sample. Consequently, the current study does not weaken confidence in the published findings, but rather, suggests that substance use to cope may be associated with less specific goals at a lower level of clinical severity. The implication is that future studies with clinical samples should measure substance use to cope to determine if this characteristic continues to be the strongest unique associate of reduced goal specificity. This work would provide greater clarity concerning the aetiology of goal specificity in the comorbidity of mental health and substance use problems.

It is worth noting that there was no association between self-rated goal quality and experimenter-coded goal specificity. This finding is consistent with a recent experiment in which no correlation was found between self- and experimenter-ratings of future events in individuals with alcohol use disorder, in contrast to healthy controls in which these measures did correlate (Noël et al., 2022). One possible explanation is that individuals with more severe symptoms lack insight into the reduced quality of their goals, so self- and experimenter-rated indices of goal quality diverge as level of severity increases. A recent neuroimaging study may provide some support for this, showing that reported subjective experience (i.e., vividness) and produced objective contents (i.e., the amount of episodic details) when imagining future events were disassociated in activating different brain networks (Thakral et al., 2020). Alternatively, this discrepancy between self- and experimenter-ratings may reflect difficulties in expressing goals in language, such that individuals rate their imagined goal rather than their written goal, which the experimenter rates (Hallford et al., 2020). Generally, many questions remain about the optimal methods for quantifying goal generation as a variety of protocols have been used but psychometric evaluation is lacking (Miloyan & McFarlane, 2019). Given increasing research interest in goal generation, it would be worthwhile to devote efforts to develop and validate measurement instruments, which might help unravel the apparent divergence between self- and experimenter-ratings, enable direct comparison between studies, and more sensitively detect which individual characteristics are most uniquely linked to goal generation.

It remains unknown what mechanism underlies the unique association that drinking to cope has with less specific and bleaker goal descriptions. One possibility is that individuals who drink to cope possess a biologically determined neurocognitive trait which is accompanied by reduced capacity to generate personal goals. For instance, emotion dysregulation, including lack of emotional clarity, limited emotional strategies and distress intolerance, has been associated with alcohol use to cope in college drinkers (Aurora & Klanecky, 2016; Veilleux et al., 2014; Williams et al., 2015). However, social disadvantage could also be the root cause. Neighbourhood disadvantage and low socioeconomic status have been associated with both reduced future orientation (Lau et al., 2017; Xiao et al., 2021) and drinking to cope (Brenner et al., 2013; Martin et al., 2019). However, an aetiological model that plausibly links these constructs into a multistage causal risk pathway simply does not exist, as the role of goal generation in psychopathology is a relatively new field. Nevertheless, there is evidence that training increased capacity to represent future goals/events may play a role in promoting recovery from substance use problems. For instance, training individuals to vividly imagine positive future events has been shown to reduce demand and craving for cigarettes in heavy smokers (Athamneh et al., 2021), cannabis use in cannabis users (Sofis et al., 2021), alcohol demand in adults with alcohol use disorders (Meshesha et al., 2020), and self-reported drinking to cope with negative affect in hazardous student drinkers (Shuai et al., 2021, 2022). The implication is that goal generation is not just an epiphenomenon, but may play a key role in substance use to cope and substance use problems.

Several limitations need to be considered in the current study. The sample was largely female (84%), so the associations observed might not be generalizable to male samples, and the design is sub-optimal for testing gender differences. Future research should address this with a larger, gender balanced sample. Another limitation is lack of a control task where participants write about either negative or neutral future goals, or past events, to test whether drinking to cope is uniquely linked to reduced positive future goal generation or simply capacity for written description. In the present study, total word count was positively correlated with experimenter-coded specificity (*r* = .44, *p* < .001), suggesting word production and goal specificity were linked. However, drinking to cope was not uniquely associated with reduced word count, suggesting this was not responsible for the reduced specificity of their goals. Nevertheless, future studies must include additional control conditions to isolate the precise dimensions of linguistic description that is reduced in individuals who drink to cope.

To conclude, the current study provided initial evidence that drink to cope is uniquely associated with non-specific and bleak future goals in young adult hazardous drinkers. In contrast with previous findings, the current study found no unique associations between capacity to generate future goals and internalizing symptoms or alcohol dependence severity, which may be due to the recruitment of sub-clinical sample. Despite a female-dominant sample and lack of control condition to test basic linguistic capacity, the current work at least indicates that substance use to cope should be routinely collected in studies of goal generation and future orientation to examine the unique associations between these characteristics, to develop a comprehensive understanding on the aetiology of goal generation in the comorbidity of mental health and substance use problems.

## Electronic Supplementary Material

Below is the link to the electronic supplementary material.

Supplementary Material 1

## Data Availability

The data will be deposited within the University of Exeter repository ORE.
