# Supplementary material for: Drinking to Cope is Uniquely Associated with Less Specific and Bleaker Future Goal Generation in Young Hazardous Drinkers
Source: J Psychopathol Behav Assess. 2023 Feb 27;45(2):403–14. doi: 10.1007/s10862-023-10032-0 (PMC10198914; doi:10.1007/s10862-023-10032-0)
Supplement: Supplementary file 1 — Supplementary Material 1 [file 10862_2023_10032_MOESM1_ESM.docx]

## Supporting information – Full Goal Coding Scheme

**Goal specificity**

Follows Scoring system of Dickson & MacLeod (2004).

*Note: Clarification from discussions between coders:

*To code as 2 (Specific): Time, Place or Location must be present – or a specific reference to individuals close to you:

e.g., “Those close to me”; “My sister”; “My parents”

*References to COVID-19, Quarantine and Lockdown count as a specific time aspect.

*To code as 1 (Moderate): The goal must be specific in the way it is operationalised:

e.g., “Reach level 7 on guitar”; “Get above 60% in Cognition and Development”; “Do HIIT training”.

**Code 2 for specific:** A goal response was deemed specific if it described a future aspiration with a particular target feature and also included at least one of the following aspects: place, time, or people. N.B. If the place, time, or people detail is very vague (e.g., “the world”, “sometime” or simply “people”, the goal should not be coded as specific.

**Code 1 for moderate:** A moderate response included a specific target feature.

**Code 0 for general:** A general response represented a global aspiration rather than a specific target feature or unique experience."

Examples from Dickson & MacLeod (2004):


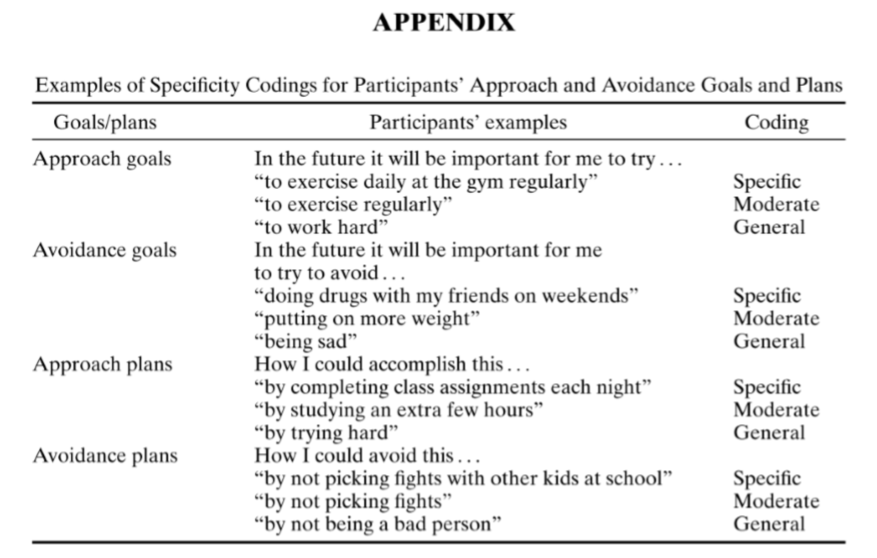


**Tricky examples:**

• Make friends [involves people, but “friends” is too vague – MODERATE]

• Get married at some point before I die [“before I die” relates to a time, but this is too vague, doesn’t add any information – MODERATE]

• Travel abroad [“abroad” does relate to place but it is not specific enough – MODERATE]

• Having a happy family [“family” entails people, but too vague; not clear if talking about existing family members, or some hypothetical future family – MODERATE]

• Live in another country [“another country” is too broad to be counted as a specific place – MODERATE]

• Meet new people [obviously includes “people”, but too broad – MODERATE]

• Find a job [quite vague, but more specific than a global aspiration – MODERATE]

• Get through the next month [time detail but not specific target feature – MODERATE]

• Be in a healthy marriage [target feature but not specific people detail – MODERATE]

• Find a girlfriend [“a girlfriend” is not a specific people detail – MODERATE]

• Live in Portugal [although “live” is quite vague, there is a specific place detail – SPECIFIC]

• Set up a company in Europe [“Europe” is specific enough to be counted as a place feature – SPECIFIC]

• Make some friends who also play tennis [specifies the “type” of friends – SPECIFIC]

• See my friends [“my friends” probably refers to specific people in this person’s life – SPECIFIC]

• Be good to my children [reference to specific people – SPECIFIC]

• Get a job after my degree [“after my degree” is a time detail – SPECIFIC]
